# The clinical significance of serum and bronchoalveolar lavage inflammatory cytokines in patients at risk for Acute Respiratory Distress Syndrome

**DOI:** 10.1186/1471-2466-4-6

**Published:** 2004-08-17

**Authors:** Demosthenes Bouros, Michael G Alexandrakis, Katerina M Antoniou, Panagiotis Agouridakis, Ioannis Pneumatikos, Stavros Anevlavis, Athanasia Pataka, George Patlakas, Nikolaos Karkavitsas, Despina Kyriakou

**Affiliations:** 1 Department of Pneumonology and Intensive Care Unit, University General Hospital of Alexandroupolis, Alexandroupolis, Greece; 2 Department of Hematology, University General Hospital of Heraklion, Heraklion, Greece; 3 Department of Pneumonology, University General Hospital of Heraklion, Heraklion, Greece; 4 Intensive Care Unit, Rethymnon General Hospital, Rethymnon, Greece; 5 Department of Nuclear Medicine, University General Hospital of Heraklion, Heraklion, Greece; 6 Department of Hematology, University General Hospital of Larissa, Larissa, Greece

**Keywords:** ARDS, cytokines, bronchoalveolar lavage, outcome

## Abstract

**Background:**

The predictive role of many cytokines has not been well defined in Acute Respiratory Distress Syndrome (ARDS).

**Methods:**

We measured prospectively IL-4, IL-6, IL-6 receptor, IL-8, and IL-10, in the serum and bronchoalveolar lavage fluid (BALF) in 59 patients who were admitted to ICU in order to identify predictive factors for the course and outcome of ARDS. The patients were divided into three groups: those fulfilling the criteria for ARDS (n = 20, group A), those at risk for ARDS and developed ARDS within 48 hours (n = 12, group B), and those at risk for ARDS but never developed ARDS (n = 27, group C).

**Results:**

An excellent negative predictive value for ARDS development was found for IL-6 in BALF and serum (100% and 95%, respectively). IL-8 in BALF and IL-8 and IL-10 serum levels were higher in non-survivors in all studied groups, and were associated with a high negative predictive value. A significant correlation was found between IL-8 and APACHE score (r = 0.60, p < 0.0001). Similarly, IL-6 and IL-6r were highly correlated with PaO2/FiO2 (r = -0.27, p < 0.05 and r = -0.55, p < 0.0001, respectively).

**Conclusions:**

BALF and serum levels of the studied cytokines on admission may provide valuable information for ARDS development in patients at risk, and outcome in patients either in ARDS or in at risk for ARDS.

## Background

Acute respiratory distress syndrome (ARDS) is characterized by respiratory failure of acute onset as a result of acute lung injury (ALI) either directly or indirectly via the blood. The main characteristics of the syndrome are diffuse inflammation and increased microvascular permeability that cause diffuses interstitial and alveolar oedema and persistent refractory hypoxemia [1]. Although a variety of insults may lead to ARDS, a common pathway may probably result in the lung damage [2-4]. A complex series of inflammatory events have been recognized during the development of ARDS but the exact sequence of the events remains unclear. Leukocyte activation and free radical release, proteases, arachidonic acid metabolites, inflammatory and anti-inflammatory cytokines results in the increased alveolar-capillary membrane permeability [5-7].

Cytokines are produced in the lung by local resident cells such as alveolar macrophages, lung epithelial cells, and fibroblasts or by cells such as neutrophils, lymphocytes, monocytes and platelets as a response to local or systemic injury [8-12]. Cytokines involved in the early phase of inflammatory response, such as IL-1, IL-2, IL-6, IL-8, [8,13,14] are secreted in response to injurious agents.

Inflammatory cytokines are of critical importance in the pathophysiology of septic shock, a condition frequently leading to ARDS [15]. It has been hypothesized that the inability of lung to repair after ALI is due to a persisted inflammatory stimulus [16].

Predictive levels of inflammatory cytokines (IL-1, IL-2, IL-6, IL-8) for ARDS development in at risk patients have been reported with controversial results [5,7,11,15,16]. Cut-off values above which ARDS development occurs in at risk patients have been also reported for IL-4 and IL-10 [16]. Schutte et al [17] compared ARDS to pneumonia and cardiogenic pulmonary oedema patients and found higher IL-6 and IL-8 values in ARDS compared to remaining populations. A systematic study of the role of all main inflammatory cytokines at the same time in the pathogenesis and development of the ARDS has not been undertaken.

The purpose of this study is to evaluate the role of inflammatory cytokines IL-4, IL-6, IL-6r, IL-8, and IL-10 in serum and bronchoalveolar lavage (BALF) as possible prognostic indicators for the development, severity, and outcome of patients with ARDS or at risk for ARDS.

## Methods

### Patients

We studied prospectively 59 consecutive patients who were admitted in our Intensive Care Units (ICU) (Table 1). The first group (group A) included 20 patients fulfilling the criteria of ARDS [1]. All these patients were supported mechanically for their respiratory failure. The second group (group B) included 12 patients on mechanical respiratory support who had at least one condition from those suggested by Fowler et al [2] as risk factors for ARDS development. All patients in this group developed ARDS within 48 hours. The third group (group C) included 27 patients on high risk for ARDS development who never developed ARDS (Table 1).

**Table 1:** Clinical features of the studied population on admission.

Group	N	Sex	Age (yr)	Diagnosis	PaO2/FiO2	APACHE II
A	20	M = 14	53 ± 19	Trauma 9	121 ± 10	19.8 ± 1.4
		F = 6		Pneumonia 3		
				Sepsis 2		
				Transfusion 2		
				Pancreatitis 2		
				Intoxication 1		
				Burns 1		
B	12	M = 8	56 ± 20	Sepsis 3	239 ± 30	20.5 ± 1.3
		F = 4		Pneumonia 4		
				Trauma 4		
				Pancreatitis 1		
C	27	M = 21	49 ± 18	Sepsis 12	276 ± 16	16.0 ± 1.1*
		F = 6		Trauma 5		
				Pneumonia 2		
				Transfusion 2		
				Intoxication 2		
				Arrest 2		
				Pancreatitis 2		
D	33	M = 20	36 ± 16			
		F = 13				

M = male, F = female, burns = >40% of the body surface. * p < 0.05 group A vs groups B and C.

For patients' classification, the following criteria were employed: 1. The ARDS criteria of the American-European Consensus Conference on ARDS (1): a. acute onset, b. bilateral chest radiographic infiltrates, c. pulmonary artery occlusion pressure of ≤18 mm Hg, or no evidence of left atrial hypertension, and d. impaired oxygenation regardless of the PEEP concentration, with a PaO_2_/FiO_2 _ratio of ≤ 300 torr for ALI and ≤ 200 torr for ARDS. 2. The high-risk criteria for ARDS development according to Fowler et al [2]). 3. The criteria for pneumonia according to EPIC study [18], and 4. The criteria for septic syndrome according to Bone et al [19]. Acute Physiology and Chronic Health Evaluation-II (APACHE II) scoring system was used for grading the disease severity [20]).

The main clinical features of the patients are shown in Table 1. The protocol was approved by the Ethics Committee of our institutions.

After admission to the ICU blood samples were obtained from a central venous line within 2 hours. APACHE II score and PaO_2_/FiO_2 _values were obtained at the time of sample collection. The blood was collected in a heparinized vacutainer tube and kept immediately at 4°C. After centrifugation at 1500 g at 4°C, the plasma was kept at -80°C until the measurement. Immediately after blood collection BALF was obtained by fiberoptic bronchoscopy. The fluid was filtered through nylon net to remove the mucous secretions, and centrifuged at 500 g for 10 min to remove cells. The supernatant was kept in cryotubes at -80°C in aliquots of 0.5 ml. The method of micro-lavage was used as described previously [21]. The following criteria were used for an acceptable sample: a. The procedure should be shorter than 1 min, while the time of saline staying into the lungs should be less than 20 sec, b. recovery of more than 50% of the saline used for the lavage, c. absence of obvious blood contamination in the BALF, and d. the level of urea in the BALF should be more than 0.4 mmol. The urea level was used as an index of BALF dilution [21]. To check the accuracy of the method, two subsequent lavages were taken in 8 patients and all the studied parameters in the two samples did not differ significantly.

### Measurement of the plasma cytokines

The assay method for cytokine measurement was the same for blood and BALF samples. Determination of plasma cytokines was done with solid phase enzyme-linked immunosorbent assay (ELISA) methodology based on the quantitative immunometric sandwich enzyme immunoassay technique [22]. Reagents for the studied cytokines were obtained from several sources (kits of R&D systems, Inc. Minneapolis, MN, USA, for IL-6R, kits of Genzyme Diagnostics, Cambridge, MA, USA for IL-8, IL-10, and RIA kits of Amersham, Buckinghamshire, UK, for IL-4, IL-6) were used according to manufacturer's instructions. Intra-assay and inter-assay reproducibility was checked and found more than 90%. To calculate the dilution factor of the BALF, urea values in the plasma and BALF were used because this low molecular weight substance is found to be in the body fluids at the same concentration as in the blood.

### Statistical analysis

Data analysis was carried out using SPSS 8.0 statistical software (SPSS Inc., Chicago, IL). Results are expressed as mean ± 1SD, or median (range), unless otherwise indicated. The Mann-Whitney non-parametric test was used to compare the mean values of the cytokines in the blood and BALF in the various groups. Receiver-operating characteristic (ROC) correlation was used to find the optimal cut-off values of the studied cytokines for ARDS development in patient at risk and survival of the patient population [23]. For tests of association, we calculated Spearman's correlation coefficient. A p value <0.05 was considered to be statistically significant.

## Results

There was no significant difference in the mean age of the patients among the three studied groups. Using APACHE-II score to determine the severity of the disease, significant difference between group A and group C (p = 0.04) and group B and C (p = 0.045) was found, and not between groups A and B (p = 0.06). The mean time of staying in the ICU did not differ among the three groups.

### Predictive capabilities of BALF mediators for onset of ARDS

The mean values (+/- SD) of the measured cytokines in BALF and serum in the three studied patient groups are shown in Table 2. A significant difference was found for BALF IL-6r, which was higher in group A than in groups B and C (p < 0.0001). Similarly, BALF IL-6 was higher in groups A and B compared to C (p < 0.01).

**Table 2:** Mean (+/-SD) BALF and serum levels of studied cytokines in the three groups of patients.

	Group A (n = 20)		Group B (n = 12)		Group C (n = 27)	
	
Cytokines (pg/mL)	BALF	Serum	BALF	Serum	BALF	Serum
IL-4	260 ± 181	158 ± 68	284 ± 119	95 ± 35^1^	242 ± 147	83 ± 68^1^
IL-6	538 ± 432^2^	388 ± 324^3^	1135 ± 1382^2^	505 ± 217	318 ± 446	313 ± 373^3^
IL-6r	180 ± 52	30 ± 25	80 ± 37^4^	34 ± 26	73 ± 22^4^	39 ± 45
IL-8	480 ± 222	3525 ± 1523^5^	492 ± 165	3543 ± 2265^5^	467 ± 179	2553 ± 2824
IL-10	62 ± 24	117 ± 60	111 ± 98	177 ± 117	73 ± 50	118 ± 84

^1^p < 0.0001 versus group A, ^2^p < 0.01 versus group C. ^3^p < 0.05 versus group B. ^4^p < 0.0001 versus group A. ^5^p < 0.0001 versus group C.

### Predictive capabilities of serum mediators for onset of ARDS

Serum levels of IL-4 were higher in group A compared to groups B and C (p < 0.0001). Serum IL-6 was higher in group B compared to group A and C (p < 0.05). Serum levels of IL-8 was higher in group A and group B compared to group C (p < 0.0001) (Table 2). Predictive values for ARDS development in at risk patients (groups B and C) for BALF and serum IL-6 are shown in Table 3. IL-6 negative predictive values for ARDS development were 100% and 95% for BALF cut off value of 195 pg/ml and serum cut off value of 255 pg/ml, respectively.

**Table 3:** BALF and serum IL-6 predictive values for ARDS development in patients at risk (n = 39, groups B+C).

	Criterion	PPV	NPV	Sensitivity	Specificity	Prevalence	95% CI
BALF							
							
IL-6, *(pg/mL)*	>195	44	100	100	62	24	0.62–0.91
Serum							
							
IL-6, *(pg/mL)*	>255	44	95	88	65	24	0.60–0.90

PPV: positive predictive value, NPV: negative predictive value. CI: confidence interval

### Predictive capabilities of BALF mediators for survival of ARDS

Mean (SEM) values in BALF and serum of the studied mediators in the survivors and non-survivors (groups A+B+C) are shown in Table 4. BALF levels of IL-6, IL-6r and IL-8 were significantly higher in those who did not survive (p < 0.05, p < 0.05 and p < 0.0001, respectively).

**Table 4:** Mean (SEM) BALF and serum levels of the measured cytokines in all patients (Groups A+B+C) according to survival.

	BALF		Serum	
Cytokines (pg/ml)	survivors (n = 30)	non-survivors (n = 29)	survivors (n = 30)	non-survivors (n = 29)

IL-4	262 ± 188	247 ± 108	72 ± 51	154 ± 68***
IL-6	313 ± 427	743 ± 877*	218 ± 191	530 ± 389***
IL-6r	94 ± 52	129 ± 69*	18 ± 19	53 ± 42**
IL-8	340 ± 109	621 ± 144***	1269 ± 830	4957 ± 1965***
IL-10	69 ± 37	82 ± 70	70 ± 16	188 ± 84***

* p < 0.05, ** p < 0.001, *** p < 0.0001 versus survivors group.

Patients with ARDS (group A) who did not survive had significantly higher BALF levels of IL-8 (p < 0.0001) and significantly lower BALF levels of IL-10 (p < 0.001) (Table 5). Patients at risk (groups B and C) who did not survive had significantly higher BALF levels of IL-8 (p < 0.0001) (Table 6). IL-6, IL6-r and IL-8 BALF concentration cut off predictive values for surviving patients are shown in Table 7. BALF IL-8 was also elevated in patients of group C who died (p < 0.0001) (Table 8).

**Table 5:** Mean (SEM) BALF and serum levels of cytokines in ARDS patients according to survival (group A).

	BALF		Serum	
	
Cytokines (pg/ml)	Survivors (n = 6)	Non-survivors (n = 14)	Survivors (n = 6)	Non-survivors (n = 14)
IL-4	352 ± 291	224 ± 115	155 ± 63	160 ± 73
IL-6	361 ± 238	606 ± 476	213 ± 140	455 ± 353
IL-6r	180 ± 38	180 ± 59	24 ± 24	31 ± 25
IL-8	218 ± 79	581 ± 167***	2028 ± 700	4100 ± 1353*
IL-10	80 ± 12	55 ± 24**	63 ± 11	138 ± 58*

*p < 0.05, ** p < 0.001, *** p < 0.0001 versus survivors group.

**Table 6:** Mean (SEM) BALF and serum levels of cytokines in at risk patients who developed or not ARDS according to survival (Groups B and C).

	BALF		Serum	
	
Cytokines (pg/ml)	Survivors (n = 24)	Non-survivors (n = 15)	Survivors (n = 24)	Non-survivors (n = 15)
IL-4	242 ± 160	271 ± 100	53 ± 21	147 ± 65***
IL-6	302 ± 463	890 ± 1178	219 ± 203	612 ± 423**
IL-6r	74 ± 29	74 ± 18	17 ± 18	77 ± 44***
IL-8	368 ± 96	664 ± 105***	1097 ± 770	5885 ± 2151***
IL-10	67 ± 40	111 ± 91	72 ± 17	243 ± 74***

* p < 0.01, ** p < 0.001, *** p < 0.0001 versus survivors group.

**Table 7:** Predictive BALF and serum levels (pg/ml) for surviving patients of all groups (n = 59)

	Criterion	PPV	NPV	Sensitivity	Specificity	95 % CI
	
BALF						
IL-6	299	68	70	68	70	0.57–0.83
IL-6r	101	65	63	52	74	0.52–0.79
IL-8	481	96	90	88	96	0.85–0.99
Serum						
IL-4	84	81	100	100	78	0.77–0.96
IL-6	160	69	94	96	59	0.69–0.92
IL-6r	18	76	78	76	78	0.66–0.89
IL-8	2340	92	96	96	93	0.90–0.99
IL-10	98	96	93	92	96	0.84–0.99

PPV: positive predictive value, NPV: negative predictive value, CI: confidence interval.

**Table 8:** Mean (SEM) BALF and serum levels of cytokines in at risk patients who did not develop ARDS (group C)

	BALF		Serum	
	
Cytokines (pg/ml)	Survivors (n = 19)	Non-survivors (n = 8)	Survivors (n = 19)	Non-survivors (n = 8)
IL-4	242 ± 169	243 ± 69	51 ± 22	169 ± 78**
IL-6	297 ± 497	374 ± 285	199 ± 197	620 ± 559*
IL-6r	70 ± 25	80 ± 7	17 ± 19	98 ± 43**
IL-8	377 ± 95	711 ± 102***	1102 ± 810	6496 ± 2543***
IL-10	69 ± 43	85 ± 69	73 ± 17	240 ± 70***

*p < 0.05, **p < 0.001, ***p < 0.0001 versus survivors group.

### Predictive capabilities of serum mediators for survival of ARDS

Cytokine concentration cut off predictive values for surviving patients are shown in Table 7. All studied mediators were found at higher levels in the serum of non-survivors (p < 0.001 to p < 0.0001). In patients at risk (groups B and C) who did not survive all serum mediators were significantly elevated (p < 0.001 to p < 0.0001) (Table 6). Serum levels of all the studied molecules were increased in all patients that did not survive (p < 0.05 to p < 0.0001) (Table 8).

In survivors BALF/serum ratios were significantly higher for IL-4, IL-8, IL-10 (p < 0.0001, p < 0.001 and p < 0.0001, respectively), due to lower serum levels and not to higher BALF levels.

### Correlations of the studied cytokines

**Figure 1:**
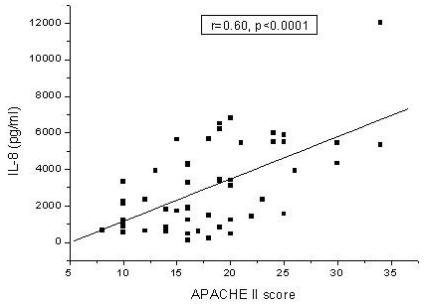
Positive strong correlation of serum levels of IL-8 to APACHE II score (Spearman's rank order correlation coefficient).

Furthermore, the serum levels of all studied mediators were significantly correlated to APACHE II score. Serum IL-8 exhibited the strongest correlation with APACHE II score (Figure 1). The level of IL-8 in the BALF were found to be significantly correlated to APACHE II score (r = 0.60, p < 0.0001).

**Figure 2:**
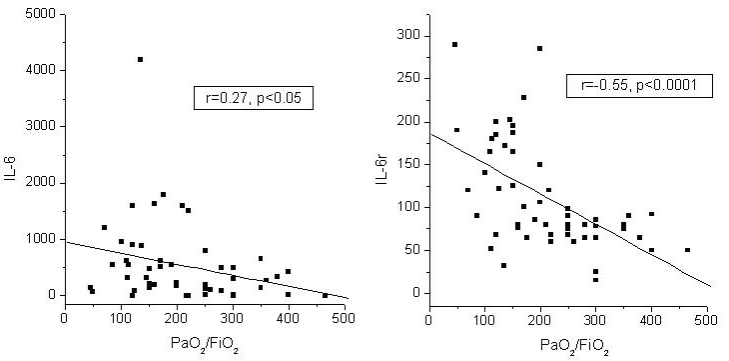
Negative correlation of BALF levels of IL-6, and IL6 receptor to PaO_2_/FiO_2 _ratio (Spearman's rank order correlation coefficient).

PaO_2_/FiO_2 _ratio was significantly correlated to the BALF levels of IL-6 and IL-6r (r = -0.27, p < 0.05; r = -0.55, p < 0.0001; respectively) (Figure 2) and to serum levels of IL-4 (r = -0.36, p < 0.05).

## Discussion

We designed this study in order to explore factors that could have prognostic value for the development, the severity, and the outcome of patients with ARDS and at risk for ARDS.

### Prediction of ARDS development

We observed that BALF levels of IL-6r were significantly higher in group A than in groups B and C (p < 0.0001), while no difference was observed in serum levels among the three groups of patients. Interestingly, the BALF and serum levels of cytokine IL-6 were significantly higher in patients at risk who developed ARDS (group B) compared to the other two groups. This observation differs from previous studies [24,25], probably reflecting the different patient population from our study. However, our results are in agreement with previous reports regarding the luck of its prediction capacity for ARDS onset [25,26], since both BALF and serum IL-6 levels, showed a low positive predictive value according to the ROC analysis.

Patients of group A and group B had higher serum levels of the inflammatory cytokine IL-8 compared to the patients of group C, but neither serum nor BALF IL-8 levels were predictive for ARDS development. In two studies, Miller et al, [27] found that IL-8 in BAL at the beginning of ARDS was highest in patients who died, and Donnelly et al [28] found that IL-8 was highest in patients at risk for ARDS who later developed ARDS. Unfortunately, subsequent studies have found that IL-8 does not predict outcome either at the outset or during the course of ARDS [5]). Meduri et al [16] found that all cytokines measured remained high during the course of ARDS in patients who died. The importance of considering anti-inflammatory constituents of BALF is shown by Donnelly et al [28] who found that patients with ARDS who died had significantly lower initial concentrations of IL-10 in BAL than patients who lived. Parson et al [29]) studied serial levels of IL-1ra and IL-10 in patients who were identified as being at risk for the development of ARDS. Initial IL-1ra levels were significantly higher (p < 0.0001) in the patients than in normal control subjects. Similarly, IL-10 levels were increased in patients compared with normal control subjects but did not predict the development of ARDS. Like IL-1ra levels, initial IL-10 levels were significantly higher (p = 0.005) in patients who died compared with survivors.

However, in other studies increased levels of IL-4, and IL-10 in serum and/or BALF were found to have beneficial effect in pre-ARDS patients [13,14]. Thus, the heterogeneity of patients in the various studies may be a reason for the contradictive results reported earlier.

### Prediction of outcome

Patients who died had significantly higher levels of IL-6, IL-6r and IL-8 in BALF than those who finally survived, while all mediators studied were significantly higher in the serum of non-survivors. The rationale for analysis of cytokine concentrations in BAL fluid is that inflammatory cytokines, like IL-6 and IL-8 are known to be produced by airway epithelial cells and activated pulmonary macrophages in response to a variety of infectious agents and other triggers of airway inflammation [30]. During ARDS, the alveolar epithelial-endothelial barrier is disrupted, and cytokines produced in the lung are released into the systemic circulation. This is believed to be a potential mechanism for the development of systemic inflammatory response syndrome [31,32]. The relationship between circulatory and pulmonary cytokines levels and outcome provides support to the hypothesis that poor outcome in ARDS is related to a persistent inflammatory process [30-33]. In addition, in agreement with our findings, bronchoalveolar concentrations of the above cytokines have been reported to be increased in patients with or at risk for ARDS [33]. As demonstrated by Meduri and co-workers, BAL fluid concentrations of IL-8 and IL-6 were significantly higher in nonsurvivors than in survivors [31]. Increased BAL levels most likely indicate intrapulmonary overproduction and not increased permeability [33]. Therefore, determination of these selected inflammatory cytokines in BAL fluid in ARDS could be of prognostic relevance [33,34]. Regarding serum levels, patients at risk (groups B and C) who died had all molecules significantly increased (Table 6), suggesting that systemic inflammatory over-response in critically ill patients may be destructive leading to multiple organ dysfunction and poor outcome. Serum levels of all the studied molecules were increased taking all patients together (groups A+B+C, Table 4) or separate (Tables 5, 6, and 8) that did not survive, suggesting that cytokinemia might reflect the severity and extension of inflammation but is not the only factor related to ARDS development. Interestingly, only IL-8 and IL-10 both in BALF and serum were higher in ARDS patients who died. These results are consistent with those of Donnelly and coworkers, who found elevated concentrations of IL-10 in BALF of 28 patients with ARDS [35]. However, our results differ from those of Armstrong and Millar, who found significantly lower concentrations of IL-10 in a small number group of patients at risk for ARDS [36]. In addition, low concentrations of IL-10 in BALF from patients with ARDS were found to be associated with increased mortality [35,37]. In contrast, all cytokines were elevated in those who died taking together all the patients at risk (groups B and C). Regarding the survival prediction, IL-8 and IL-10 showed the higher serum positive predictive value (92 and 96%, respectively), while IL-4 had the higher serum negative predictive value and sensitivity, taking together all patients.

### Relation to severity of lung injury

Regarding the relation of the studied molecules and the severity of lung injury, a negative correlation was found between BALF IL-6, and IL-6r and PaO2/FiO2. The same was true for serum IL-4 and PaO2/FiO2. All the studied molecules in the serum were positively correlated with the APACHE II score, as was BALF IL-8. It is probable that this cytokine is closely related to the extension of tissue damage and organ failure.

## Conclusions

In conclusion, our data show that the predictive role of most of the studied molecules both in serum and BALF for ARDS development is valuable. In addition, almost all of them are good predictors of outcome in these patients. Further studies with greater number of patients with various subgroups of ARDS as well as stricter grouping criteria should be designed to investigate the complex network of these molecules and their receptors in ARDS and their value as predictive factors in these patients.

## Competing interest

None declared.

## Authors' contributions

DB conceived of the study, and participated in its design and coordination and drafted the manuscript

MGA participated in the design of the study, carried out immunoassays and drafted the manuscript

KMA carried out immunoassays and drafted the manuscript

PA patients data and samples collection

IP patients data and samples collection

SA patients data and samples collection

GP Performed statistical analysis

AP patients data and samples collection

NK carried out RIA measurements

DP KMA carried out immunoassays and drafted the manuscript

All authors read and approved the final manuscript

## Pre-publication history

The pre-publication history for this paper can be accessed here:


